# Supplementary figures and images for: Transcriptome-guided parsimonious flux analysis improves predictions with metabolic networks in complex environments
Source: PLoS Comput Biol. 2020 Apr 16;16(4):e1007099. doi: 10.1371/journal.pcbi.1007099 (PMC7188308; doi:10.1371/journal.pcbi.1007099)

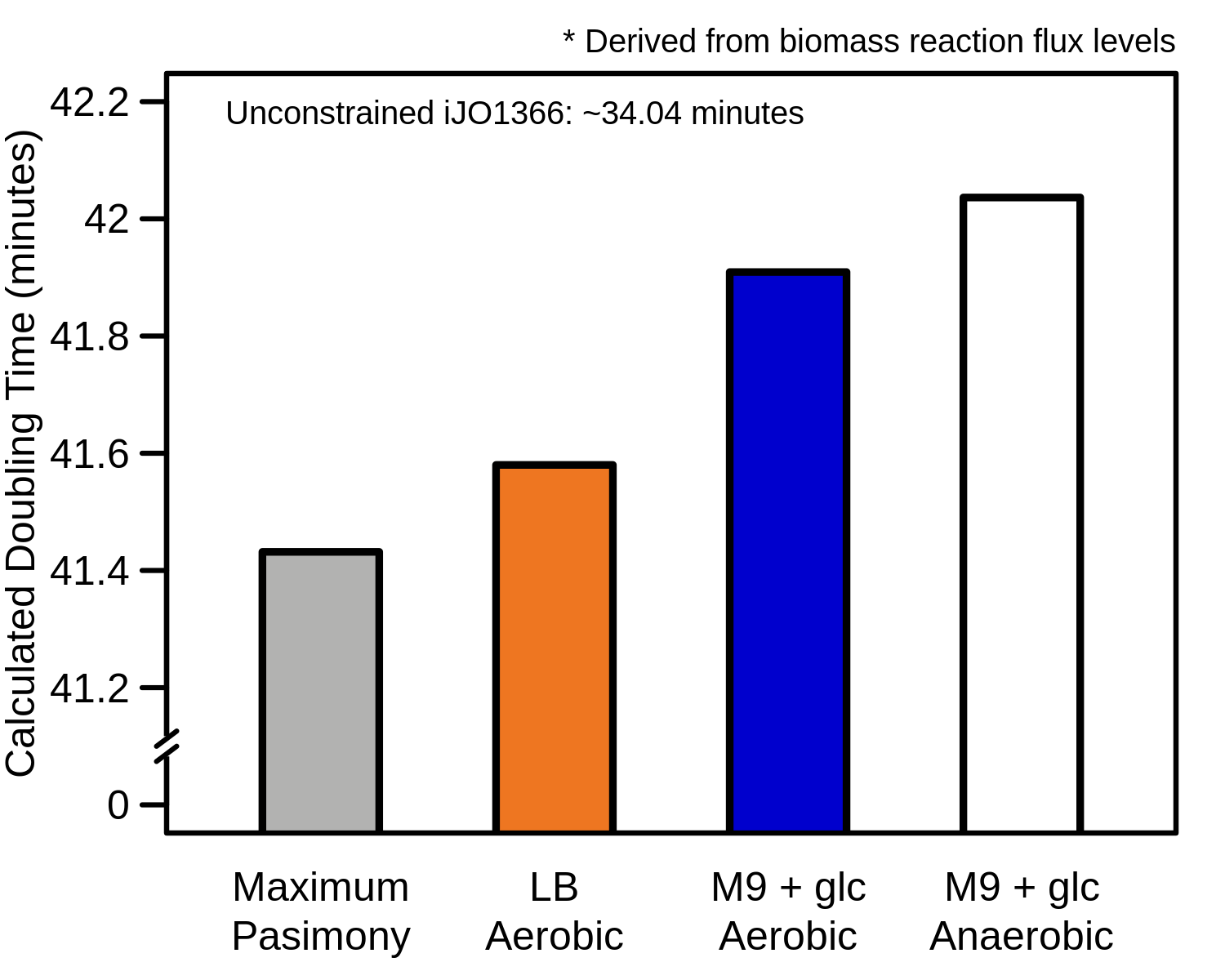

Supplement: S1 Fig — Computed doubling time from biomass reaction objective value following FBA analysis of each contextualized model (all open exchange reactions). Result from the unconstrained model is listed along the top axis. Biomass objective flux was constrained to ≧80% of the optimal flux value prior to identifying the state of Maximum Parsimony as it is during RIPTiDe contextualization. (TIF) [file pcbi.1007099.s001.tif]

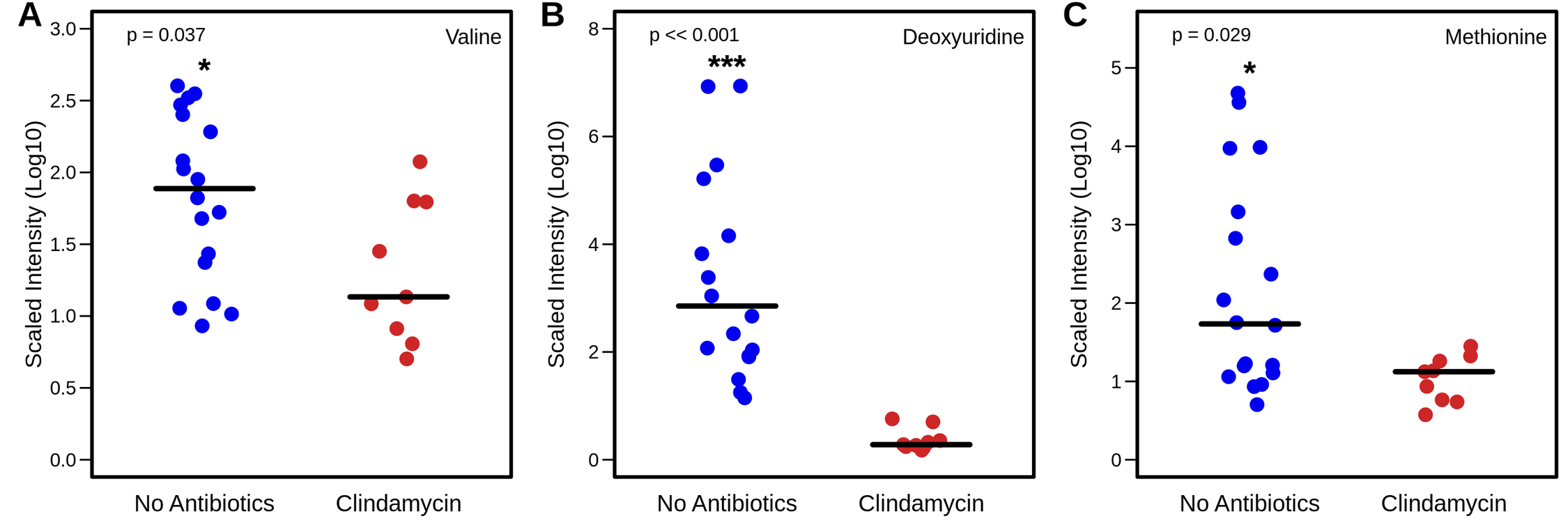

Supplement: S2 Fig — Concentrations quantified with liquid chromatography mass-spectrometry previously [3]. Comparing the metabolite content in cecal content of mice pretreated with an intraperitoneal injection of clindamycin against untreated control animals. (A) Valine, (B) Deoxyuridine, (C) and Methionine. Significant differences were determined by Wilcoxon signed-rank test. (TIF) [file pcbi.1007099.s002.tif]

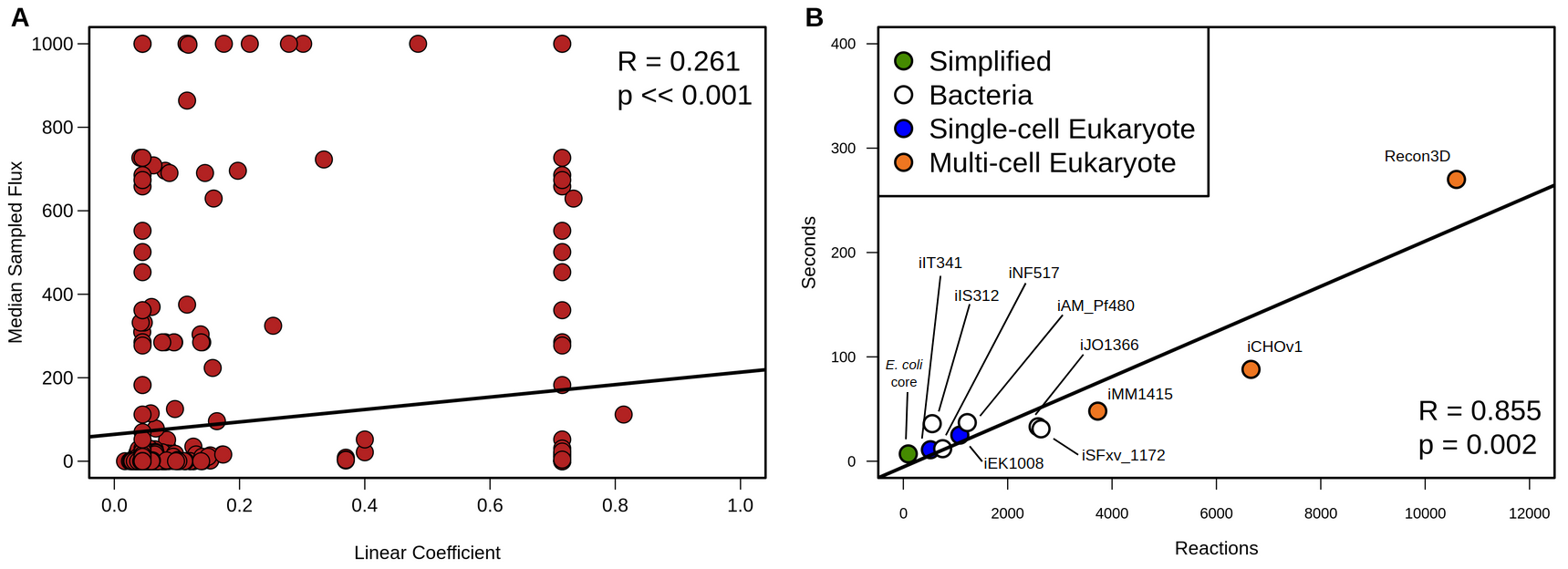

Supplement: S3 Fig — (A) Spearman correlation results between RIPTiDe linear coefficients from flux sampling and median absolute values from sampled flux ranges from in vivo transcriptome. Assigned linear coefficients are significantly correlated with their associated absolute reaction activities. Correlation results are referred to as concordance in the output of RIPTiDe. (B) Run time in seconds with increasing metabolic network reconstruction size. Using RIPTiDe, each GENRE (11 total) was integrated with a simulated transcriptome of 1.0 transcript for all genes. Multicellular eukaryotic models include mouse (iMM1415), golden hamster (iCHOv1), and human (Recon3D). The relationship between model size and RIPTiDe run time fits a linear model (p-value = 0.002). These analyses were completed using an Intel Core i7-7Y75 CPU @ 1.30GHz × 4 processor and 15.4 GB of memory. (TIF) [file pcbi.1007099.s003.tif]

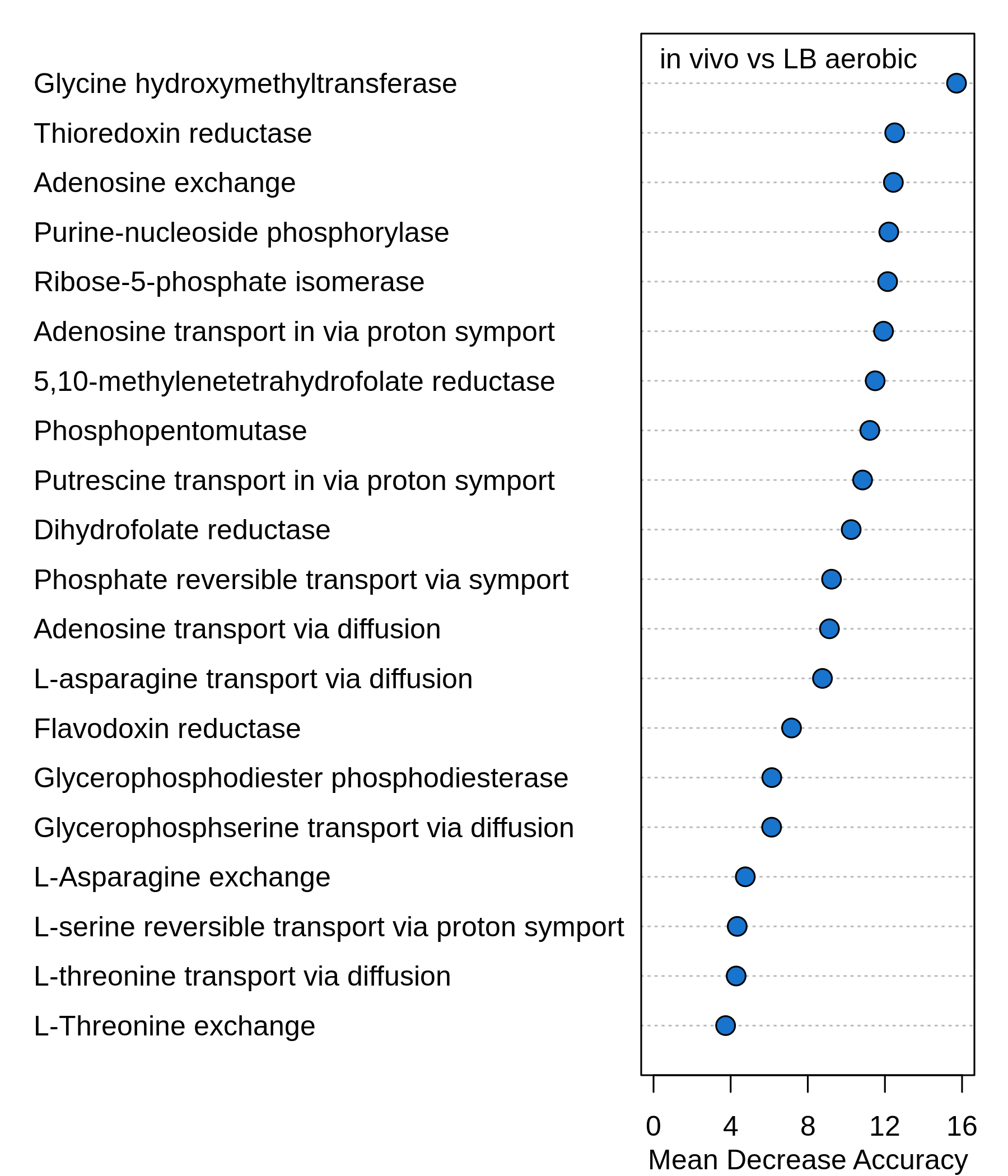

Supplement: S4 Fig — Mean Decrease Accuracy for an optimal subset of 20 reactions determined by AUC Random Forest that flux levels differentiate the distributions from shared reactions between in vivo and LB rich media RIPTiDe-contextualized transcriptomes of E. coli. (TIF) [file pcbi.1007099.s004.tif]

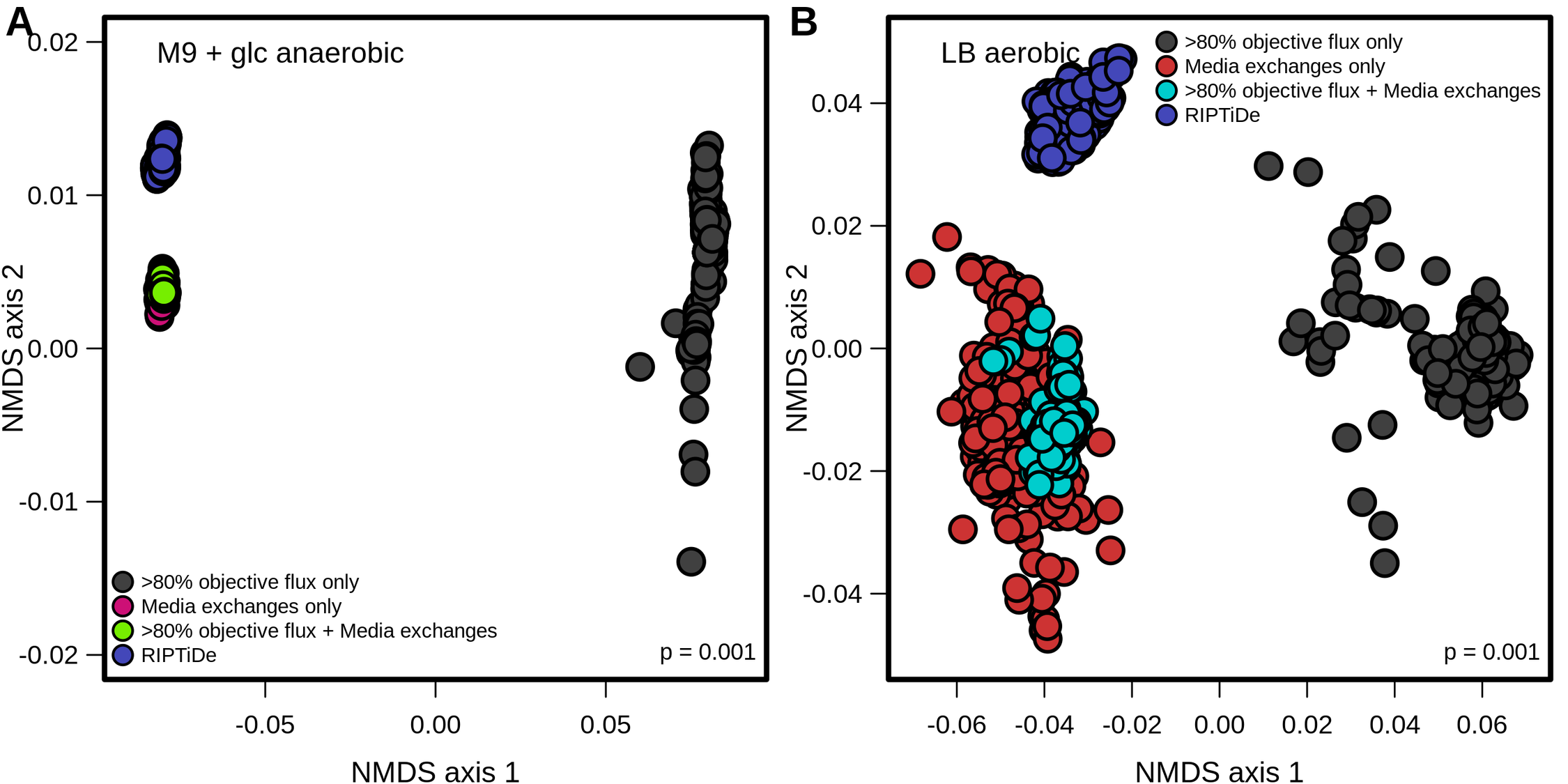

Supplement: S5 Fig — NMDS ordination of Bray-Curtis dissimilarities between flux samples for each version of iJO1366 in (A) M9+glucose anaerobic and (B) LB aerobic. Significant differences between constrained objective flux only and all other groups determined by PERMANOVA. The dissimilarity of RIPTiDe-generated flux samples in these analyses highlight context-specific metabolic patterns that were unobservable through other methods. (TIF) [file pcbi.1007099.s005.tif]
